# Host selection tendency of key microbiota in arid desert lichen crusts

**DOI:** 10.1002/imt2.138

**Published:** 2023-10-10

**Authors:** Ting‐Ting Zhang, Martin Grube, Xin‐Li Wei

**Affiliations:** ^1^ State Key Laboratory of Mycology, Institute of Microbiology Chinese Academy of Sciences Beijing China; ^2^ College of Life Sciences University of Chinese Academy of Sciences Beijing China; ^3^ Institute of Biology University of Graz Graz Austria

## Abstract

Lichen genus *Endocarpon* in biological soil crust form was chosen as a model to investigate the bacterial communities for the first time across four vertically distinct strata. Key bacterial microbiota in lichen thallus were discovered, which were gradually filtered and mainly derived from the crust soil, with clear host selection tendency. The study provided key information to better understand the homeostasis maintenance mechanism of the lichen symbiont and community assembly of desert lichen crust.

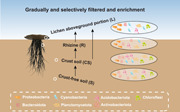

The dramatic expansion of land desertification is one of the most critical problems worldwide [1]. Deserts represent arid environments with poor soil nutrients, [2] where vascular plants have low coverage, but biological soil crusts (BSCs) have unique advantages [3]. BSCs cover 70% of arid and semiarid areas, and constitute about 40% of the Earth's terrestrial surface, [4, 5] composed of cyanobacteria, algae, lichens, microfungi, bryophytes, [4, 6, 7] among which lichens play more crucial roles such as promoting nutrient cycling and improving soil physical stability [8, 9].

Lichens are symbiotic associations of fungi (mycobiont) with algae and/or cyanobacteria (photobiont) [10]. They also include associated microorganisms, [11] such as bacteria and fungi [12, 13, 14, 15]. Fluorescence in situ hybridization studies have revealed high diversity and prevalence of bacteria in lichen thalli [16, 17]. Metagenomic analysis indicated lichen‐associated bacteria may be involved in nutrient supply, resistance against biotic and abiotic stress, photosynthesis, and detoxification and thallus degradation [12, 18]. In short, lichens might be inseparable from the associated bacteria, and can also be serve as a reservoir of bacterial species preserved in harsh environments. A lichen‐associated bacterial community can be shaped by multiple factors, such as substrate type, thallus age, and the immediate environment of the host [19, 20, 21, 22]. However, whether the potential assembly mechanisms of a lichen bacterial microbiota would be related to the lichen symbiosis and how the lichen symbiont maintained its homeostasis are poorly known.

Several studies have investigated the bacterial species composition in corticolous and saxicolous lichens, [12, 23, 24, 25, 26, 27] but rarely involved of possible source and therefore correspondingly rather hard to explore the potential relationship to the lichen symbiosis. Studies on terricolous lichens have primarily focused on community composition and physiological performance. In addition, lichen crusts for these studies were sampled within a few millimeters of the surface including sand soil, [4] irrespective of the details of lichen morphology, which could be biased for adjacent microbiota of soil particles trapped by their attachment to structures such as rhizines or tomentum [28]. Despite the increasing research focus on the lichens in biological soil crust, [29] there is a lack of adequate understanding on the community characteristics of the terricolous lichen microbiota, co‐occurrence network structures, and potential drivers [30, 31, 32].

Addressing these issues, we selected the lichen genus *Endocarpon* Hedw. considering its wide distribution in arid deserts and remarkable carbon‐ and sand‐fixation potential [33]. We collected two types of *Endocarpon* crusts (assigned to *Endocarpon adsurgens*, *Endocarpon pusillum*) in deserts of China and subdivided them into four distinct strata for the first time, that is, lichen aboveground portion (L), lichen underground portion (R), crust soil (CS), and crust‐free soil (S). We then amplified the 16S rDNA hypervariable region 4 from each stratum and sequenced them through the Illumina NovaSeqPE250 platform. We hypothesize lichen can make targeted selection of its co‐existing microbiota from the environment to maintain its own homeostasis, which is rarely affected by lichen species, so correspondingly design to (1) dissect the composition of bacterial communities of different parts of *Endocarpon* crusts, (2) elucidate the factors affecting bacterial community assemblies, (3) explore the possible source and potential function, and (4) uncover the bacterial molecular co‐occurrence network structures.

## RESULTS

### Identification of Illumina sequencing data

The *Endocarpon* crusts contained two species, that is, *E. adsurgens* Vain. (66 samples) and *E. pusillum* Hedw. (54 samples). We generated a total of 13,058,562 high‐quality sequences from these 120 samples. After discarding low‐abundance ZOTUs (<8 total counts), deduplication, and exclusion singleton, denoising reads generated 22,808 ZOTUs. 6,682,292 reads containing 21,652 ZOTUs were used for subsequent analysis after exclusion of 1156 ZOTUs assigned to no‐bacteria. The reads number was normalized to 5717 as it ranged from 5717 to 309,940 across the 120 samples, resulting in 686,040 reads were comprised finally. 376, 296, 681, and 160 ZOTUs were only found in the strata S, CS, R, and L, respectively, whereas 10,247 ZOTUs were shared (Figure S1).

### Diversity and community assembly of bacterial microbiota in the lichen crust

**Figure 1:**
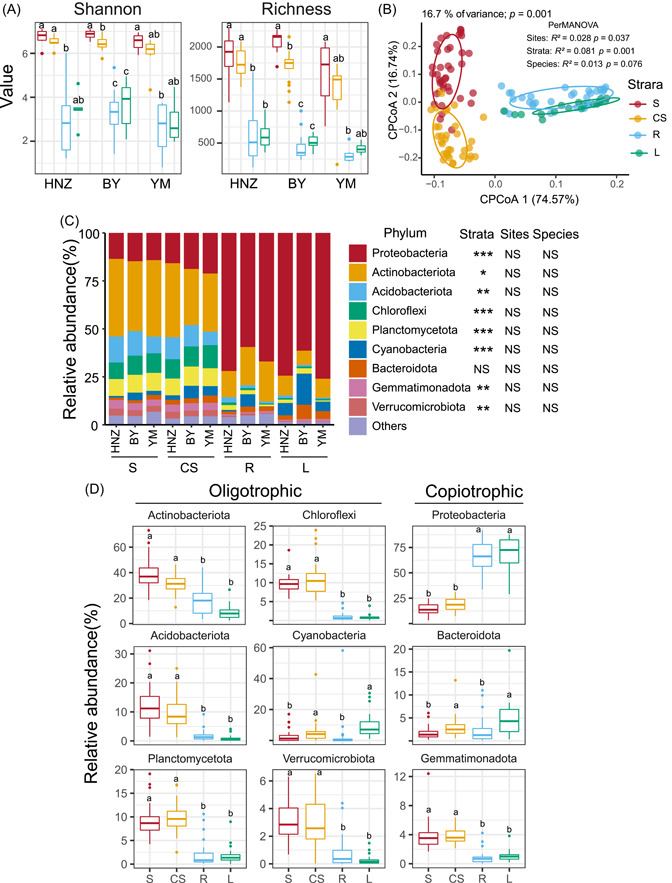
Species diversity and composition in *Endocarpon* crusts. (A) The alpha diversity indices of the richness index and Shannon index shows differences between strata. The black line inside each box represents the median value. Different lowercase letters indicate significant differences between groups (*α* = 0.05, Kruskal–Wallis test). (B) The constrained principal coordinate analysis (CPCoA) of bacterial communities based on Bray–Curtis distance dissimilarity with permutational analysis of variance (PerMANOVA), in the order of importance, the strata (*R* ^2^ = 0.081), the collection sites (*R* ^2^ = 0.028), and the lichen species (*R* ^2^ = 0.013). (C) Relative abundance of top nine bacteria phyla in different samples. Kruskal–Wallis test shows the effect of collection sites and four strata on the bacteria community composition (***p < 0.001, **p < 0.01, *p < 0.05). (D) The composition of oligotrophic and copiotrophic taxa in four strata. The black line inside each box represents the median value. Different lowercase letters indicate significant differences between groups (*α* = 0.05, Kruskal–Wallis test). BY, Baiyin City; CS, crust soil; HNZ, Tibetan Autonomous Prefecture of Hainan; L, lichen aboveground portion; NS, no significance; R, rhizine, lichen underground portion; S, crust‐free soil; YM, Yumen City.

The Kruskal–Wallis test revealed that the biocrust stratum/niche had a significant effect on the shannon index of bacterial community (*χ* ^2^ = 92.419, *p* < 0.001), but the effects of sampling sites (*χ* ^2^ = 3.0246, *p* = 0.2204) and lichen species (*χ* ^2^ = 1.4674, *p* = 0.2258) were not significant. Similarly, the influence of strata (*χ* ^2^ = 83.944, *p* < 0.001), sampling sites (*χ* ^2^ = 5.7553, *p* = 0.05627), and species (*χ* ^2^ = 2.4179, *p* = 0.12) on richness index were in the same way. These two alpha diversity indexes showed a decreasing trend in a stepwise manner from S, CS, and L to R (Dunnett's test, *p* < 0.05) (Figure 1A). These results revealed higher species diversity of bacterial microbiota in soil samples (S and CS) and lower in lichen samples (L and R).

The CPCoA of Bray–Curtis distances revealed that the four strata have taxonomically different composition (*p* = 0.001, Figure 1B). Lichen samples (L and R) were distinctly separated from soil samples (S and CS). Significant differences were also found between microbiota from different sites (adonis, *p* = 0.037), but more community variation was explained by the different strata (*R* ^2^_stratum_ = 0.081, *R* ^2^_site_ = 0.028). No significant differences in bacterial community composition between the two *Endocarpon* species (*p* = 0.076) was examined by the PerMANOVA test (Figure 1B), which supports our hypothesis that lichen microbiota are not affected by lichen species. The samples were combined for further analysis. Although the three sampling sites are thousands of kilometers away, the bacterial microbiota in L formed a close cluster (Figure 1B). The beta‐dispersion analysis also showed a decreasing trend in community dissimilarity from R, S, and CS to L (Figure S2). Distance‐decay indicated that soil samples (S and CS) had higher turnover rates than lichen samples (R and L) for bacterial community similarities over increased environmental distance (Euclidean distance of pairwise samples based on matrix of measured environmental variables) (Figure S3). The amount of interpretation of environmental distance to community similarity showed a decreasing trend from S (0.068), CS (0.043), and R (0.011) to L (0.0005). That is, the bacterial community similarities declined more sharply in the soil samples than lichen samples (Figure S3).

A slight difference was observed in the bacterial species composition at the phylum level in S and CS (Figure 1C, Figure S4), where *Actinobacteria* comprised the dominant fraction, 38.5% and 31.2% of average relative abundance, respectively, followed by *Proteobacteria* and *Acidobacteria*, with 14.3% and 11.9%, 19.0%, and 9.9%. However, significant difference occurred in R and L, as comparison, *Actinobacteriota* (32.1%), *Acidobacteriota* (9.78%), and *Verrucomicrobiota* (2.81%) were more abundant in R, while *Proteobacteria* (68.9%), *Cyanobacteria* (2.57%), and *Bacteroidota* (4.95%) were higher in L (Figure 1C,D, Figure S5).

**Figure 2:**
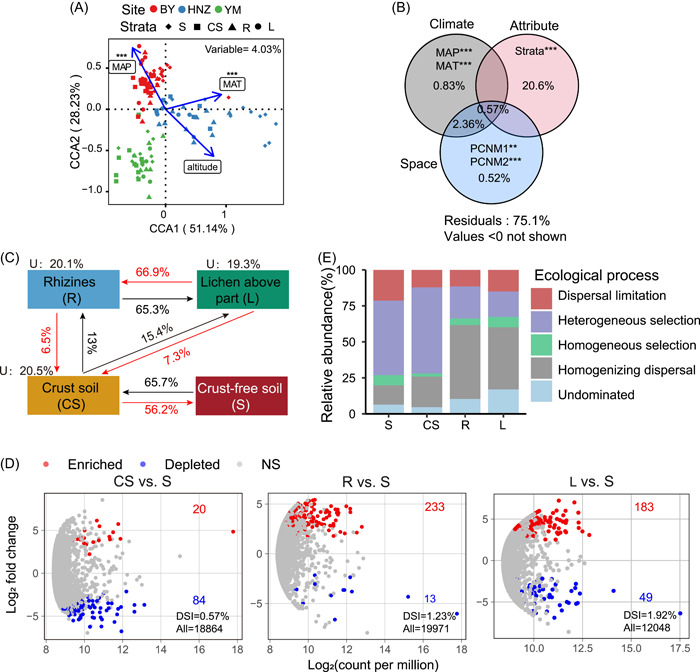
Bacterial community influenced by environmental factors, source‐tracking and assembly process in *Endocarpon* crusts. (A) Canonical correlation analysis (CCA) on bacterial community composition and selected environmental factors. (B) Venn diagrams visualize that climate factors, sample attribute and space distance possibly explain the variance of bacterial community composition (one‐way ANOVA, ***p < 0.001, **p < 0.01). Explained values <0 not shown. (C) Source‐tracking analysis showing the potential sources of *Endocarpon* crusts bacterial communities. (D) The volcano plot illustrates the enrichment and depletion patterns of the *Endocarpon* crusts bacterial microbiomes in each stratum compared with S (*p* < 0.01 and abs log_2_ fold change ≥1). The position along the *y*‐axis represents the fold‐change in abundance compared with S, and the *x*‐axis reports the average ZOTUs abundance (as counts per million, CPM). Each red point represents an individual enriched ZOTUs, a blue point represents an individual depleted ZOTUs, and a gray point represents no significance ZOTUs. All, numbers of the total ZOTUs. (E) The relative importance of five ecological processes along the four strata based on the β‐Nearest Taxon Index and Bray–Curtis based Raup–Crick Index (RCBray). DSI, dissimilarity index; U, unknown source.

The taxonomy of major bacterial compositions according to the microbial r/K spectrum were different in the strata (Dunn's Kruskal–Wallis multiple comparisons, *p* < 0.05) (Figure 1D). Oligotrophic taxa, such as *Acidobacteriota*, *Actinobacteriota*, *Planctomycetes*, *Chloroflexi*, and *Verrucomicrobia*, were more predominant in soil samples (S and CS). In contrast, the relative abundance of copiotrophic taxa, such as *Bacteroidota* and *Proteobacteria* were higher in lichen samples (L and R) (Figure 1D). At the genus level, the top 35 taxa in relative abundance were clustered into two categories according to phylogeny. The composition of high‐abundance taxa in S and CS are more similar, and can be clearly distinguished from the taxa in L. L‐associated *Cyanobacteria* were mainly predominated by *Microcoleus*, *Chroococcidiopsis* and *Craurococcus*. R had higher relative contents of the *Actinophytocola*, *Devosia*, *Bacillus*, and *Kribbella* (Figure S5). Canonical correlation analysis showed that mean annual temperature (MAT), mean annual precipitation (MAP), and altitude can explain 4.03% of the variance, while Monte Carlo permutation test showed that MAP and MAT significantly influence bacterial community composition (*p* < 0.001). Dimension one (CCA 1) clearly separates HNZ samples from others, whereas dimension two (CCA 2) separates BY and YM samples (Figure 2A). Variance partitioning analysis revealed that strata explained 20.6% of the variability in *Endocarpon* bacterial community, greater than environmental factors (MAP and MAT, total contribution 0.83%) and space distance (PCNM1 and PCNM2, 0.52%) (Figure 2B).

### Gradual selection of microbiota with host selection tendency

Source tracking suggested that lichen‐associated bacterial communities were primarily derived from crust soil and gradually filtered in the ecological strata. Specifically, L directly selected the majority of taxa from R but mainly took CS as its primary source, whereas CS primarily filtered from S (Figure 2C), indicating lichens can make targeted selection of its co‐existing microbiota from the environment. EdgeR analysis indicated that L had the greatest number (183/12,048) of specific enriched ZOTUs, and CS had the greatest number (84/18,864) of specific depleted ZOTUs (Figure 2D). ZOTUs belonged to *Thermoleophilia* were significantly enriched in R (48/233) and L (41/183) samples, but significantly depleted in CS (16/84), while *Alphaproteobacteria* were significantly enriched in CS (16/20) samples but significantly depleted in R (6/13) and L (35/49) samples (Table S1). The DSI (dissimilarity index) value gradually increased from CS (0.57%) to R (1.23%) and then to L (1.92%), indicating that the species filtration on the bacterial community gradually increased from the bottom to top in the stratum, and L had the biggest DSI value (Figure 2D, Table S1).

A null model was used in the community assembly analysis of *Endocarpon* crust. Different patterns were observed in four strata. The strength of stochastic processes (dispersal limitation, homogenizing dispersal, and undominated effects) increased in lichen samples, R (73%), and L (75%), primarily attributing to homogenizing dispersal (51% and 43%). However, deterministic processes (heterogeneous selection and homogeneous selection) affected the bacterial community assembly more in soil samples, S (59%) and CS (62%), mainly attributing to heterogeneous selection (over 50%) (Figure 2E).

### Stratum affects bacterial co‐occurrence network topology and keystone species

**Figure 3:**
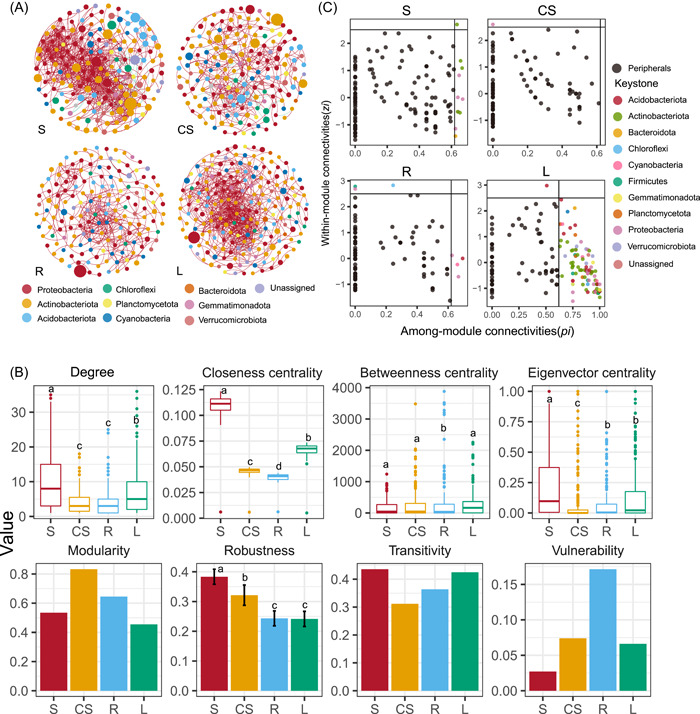
Co‐occurrence networks, putative keystone species, and topological properties analysis for bacterial communities between four strata. (A) Overview of the networks under four strata, nodes are colored for phylogenetic phylum. The size of each node is proportional to the degree of the ZOTUs; the thickness of a connection between two nodes is proportional to the value of Spearman's correlation coefficient. A red link indicates a positive correlation and a green link negative correlation. (B) Unique node level topological features of different taxa categories, especially the degree, closeness centrality, betweenness centrality, and eigenvector centrality. Other network properties consisted of modularity, transitivity, robustness, and vulnerability. Different lowercase letters indicate significant differences between groups (*α* = 0.05, Kruskal–Wallis test). The black line inside each box represents the median value. The histogram represents the mean value ± two standard variance (SD). (C) Putative keystone species in four strata networks. The colors of key nodes indicate different phyla.

A series of topological features were calculated for co‐occurrence and Erdös–Réyni random networks (Table S2). The values of average path length, average degree, and average clustering coefficient are higher than random networks, indicating a nonrandom co‐occurrence pattern and a small‐world topology in bacterial microbiota. Bacterial network patterns shifted clearly across four strata (Figure 3A, Table S2, Kruskal–Wallis test). Specifically, S had the highest network connectivity (i.e., network degree, closeness centrality, betweenness centrality, eigenvector centrality, and transitivity) (*p* > 0.05). Resistance of bacterial networks to disturbances was evaluated by random node loss, when 50% of the random nodes were removed, the remaining nodes represent network robustness. S network showed significantly higher (*p* > 0.05) robustness. As a comparison, L network had the second highest connectedness (degree), centrality (closeness centrality and eigenvector centrality), and complexity (transitivity) (*p* > 0.05). In addition, L network is characterized by the lowest modularity. In brief, the bacterial community in L was more complex and highly connected, most possibly related to sustaining its own homeostasis within lichen symbiont. R network was the simplest, and had higher vulnerability (Figure 3B). In both networks, positive correlations were dominant. The proportion of negative correlations showed a decreasing trend in a stepwise manner from CS (10.36%), S (5.31%), and L (0.93%) to R (0.88%) (Dunnett's test, *p* < 0.05) (Table S2). The majority of nodes in each network were peripherals and the main type of keystone nodes is connectors. Statistically, the phyla which took the keystone nodes in the network varied among the four strata (Table S3). Three class, *Alphaproteobacteria*, *Bacteroidia*, *Actinobacteria*, were identified as connectors in the S network, and the family *Pseudonocardiaceae* was classified as network hub. Sixteen additional class (*Thermoleophilia*, *Rubrobacteria*, *Cyanobacteria*, etc.) as connectors were in L network. *Beijerinckiaceae* was identified as the module hub in CS network, and three taxa (Ktedonobacteria, Bacillales, and Sphingomonadales) were identified as module hubs in R network (Figure 3C, Table S3).

## DISCUSSION

In this study we could show that the alpha diversity of bacteria decreased from soil toward lichen samples (Figure 1A), but because there is no description work about alpha diversity on lichen growth different partition, we can't compare our results with existing lichen literature. What the slightly related studies reported the lichen bacterial community structure at different growth stages, geographical flora, and growth substrates, and so forth [20, 34, 35, 36]. Although the bacterial microbiota is considered of importance for the holobiome, there have so far only been microscopic observations of differences between upper and lower surfaces of lichens [12]. Only one early study of rock lichens showed that the lichen thallus influenced the rock‐inhabiting bacterial communities underneath [24]. Here we closed a gap between these studies by distinguishing all vertical strata, and also assessed these differences of microbiota composition for the first time quantitatively.

Bacterial microbes showed a decrease in community similarity across environmental distances, indicating distance‐decay patterns [37, 38]. The slopes of the distance‐decay curves for taxonomic β‐diversity were higher in soil samples here (Figure S3), indicating soil samples had a higher taxonomic and phylogenetic turnover, [39] which may be due to lichen symbiosis exerting a strong host selection effect on the bacterial microbiota to sustain a relatively stable microbiota in the lichen symbiont. This also can well support our hypothesis that lichen can make targeted selection of its co‐existing microbiota from the environment to sustain its own homeostasis, which is rarely affected by lichen species.

Lichens could represent a niche for the diversification of bacteria with different functions [17, 22]. The coexisting relationships between species can fully utilize the limited resources for metabolic activities, resulting in niche differentiation [40, 41]. Copiotrophic (r‐strategists) taxa refer to species usually possessing a higher rRNA operon copy number and higher growth rate in response to the availability of resources [42, 43]. Oligotrophic (K‐strategists) microbiota possess slower growth rates and more stable populations [44, 45]. Our results demonstrated that soil samples were enriched with oligotrophic bacteria, but lichen samples were enriched with copiotrophic bacteria. Some bacterial groups enriched in R, such as *Bacillus* (Firmicutes), may participate in the degradation of the organic material applied in the soil [46, 47]. The bacterial species composition in L were similar to those reported previously on the corticolous lichen species *Schizoxylon albescens* Gilenstam, H. Döring and Wedin [22] and *Lobaria pulmonaria* (L.) Hoffm [27, 48]. and the terricolous lichen *Cladonia coccifera* (L.) Willd., [25] with the dominant bacterial microbiota fraction represented by Proteobacteria (Figure 1C). Besides, Bacteroidota were also enriched in L, which are relatively abundant in environments rich in carbon sources [49]. The increase in the relative abundances of these copiotrophic phyla found in lichen samples may be related to the increased availability of resources, which is advantageous to these fast‐growing bacterial taxa (Figure 1D, Figure S4).

We innovatively discovered that the community assembly process was different among the four strata of lichen crusts, which may get benefit from our sampling design. Heterogeneous selection (deterministic processes) played a predominant role in bacterial community assembly processes, most possibly leading to more dissimilar structures among communities [50]. Lichen samples are spatially heterogeneous, and the associated microbiota was influenced by both the host (mycobiont and photobiont) and environment, resulting in high variation in the bacterial community structure [51]). Dispersal between individuals may be limited by the binary system formed by mycobiont and photobiont, homogenizing dispersal (stochastic processes) leading to more‐dissimilar structures among communities [50].

The bacterial orders *Solirubrobacterales*, *Pseudonocardiales* and *Rubrobacterales* within *Actinobacteriota*, *Pyrinomonadales* within *Acidobacteriota*, and *Rhizobiales* within *Proteobacteria* were considered as dominant connectors in L network (Figure 3B, Table S3). R was the portion closely connected to L, and the relative dominant connectors were *Sphingomonadales* and *Rhizobiales* within *Proteobacteria*, which are also the taxa with the highest abundance. According to previous studies on culturable microorganisms, most *Actinobacteriota* are adapted to arid conditions and highly resistant to desiccation and low‐resource conditions [52, 53]. Recent studies have shown that *Actinobacteriota* are enriched during drought stress and improved the drought tolerance and growth of plants [54, 55]. *Endocarpon* generally grows in deserts with drought stress. Hence, we speculated that the abundance of *Actinobacteriota* in R would have a similar function such as lichen growth promotion by helping lichens to use nutrients from the substrate. Some other lichen‐associated bacteria (Table S3) potentially assist lichens in defending against environmental stress, [25, 56] for instance, *Sphingomonadales*, including facultatively photosynthetic taxa, and *Rhizobiales* well known for its nitrogen‐fixing symbiosis with plants [57] and participation in specific secondary metabolite and nutrient cycling in lichens [12, 27]. *Beijerinckiaceae* and *Xanthobacteraceae* of *Rhizobiales* were enriched in R and L. *Xanthobacteraceae* grows as aerobic chemoheterotrophs and nitrogen fixation is widespread, [58] while most members in *Beijerinckiaceae* produce polysaccharide capsules and are capable of fixing dinitrogen [59]. So we speculated that these bacteria existing in lichen strata might not be occasional. Besides, the acquisition of sunlight was limited in R as underground stratum, the abundant *Sphingomonadales* here indicates a possible pathway because *Sphingomonadales* consist of aerobic anoxygenic phototrophs with diverse carotenoid pigments and photosynthesis gene clusters (PGCs) [60, 61]. The source of nitrogen acquisition in chlorolichens such as *Endocarpon* is not completely clear, therefore, the abundance of *Rhizobiales* in L attracted our attention, which was also found to be common in other chlorolichens, [12] however, there is no evidence to support *Rhizobiales* help in nitrogen fixation in the chlorolichens like in the plants [57]. Our study also found that there is a relatively high amount of *Cyanobacteria* in L (Figure 1D, Figure S4). The cyanobacteria *Coleofasciculaceae*, *Chroococcidiopsaceae*, *Phormidiaceae*, and *Nostocaceae* were identified as network connectors (Table S3), whether the existence of them playing a function of nitrogen fixation in the symbiosis is still open.

## CONCLUSION

Our results demonstrated that bacterial community in lichen crusts was shaped obviously by strata, second by the collection sites, but rather than by lichen species. Furthermore, host selection had a much stronger influence on the structure of attached lichen microbiota (R and L) than the nearby soil strata. The key bacteria (i.e., *Pyrinomonadales*, *Rhizobiales*, *Solirubrobacterales*, *Sphingomonadales*) were also identified in this study. More importantly, our study demonstrated that lichen bacterial microbiota was primarily derived from CS and gradually enriched and filtered. Our study provided key information to better understand the homeostasis maintenance mechanism of the lichen symbiont.

## AUTHOR CONTRIBUTIONS

Xin‐Li Wei conceived and designed the study; Ting‐Ting Zhang collected samples, performed the experiments, analyzed the data, and wrote the manuscript draft; Xin‐Li Wei, Ting‐Ting Zhang, and Martin Grube revised the draft; all authors approved the final manuscript.

## CONFLICT OF INTEREST STATEMENT

The authors declare no conflict of interest.

## Supporting information

Supporting information.

Supporting information.

## Data Availability

The raw sequence data used in this paper have been deposited in GenBank under BioProject with accession code PRJNA873937 (https://www.ncbi.nlm.nih.gov/sra/?term=PRJNA873937). Supplementary materials (methods, figures, tables, scripts, graphical abstract, slides, videos, Chinese translated version, and update materials) may be found in the online DOI or iMeta Science http://www.imeta.science/.
