# Supplementary material for: Host selection tendency of key microbiota in arid desert lichen crusts
Source: Imeta. 2023 Oct 10;2(4):e138. doi: 10.1002/imt2.138 (PMC10989926; doi:10.1002/imt2.138)
Supplement: Supplementary file 1 — Supporting information. [file IMT2-2-e138-s001.docx]

Supporting Information to:

**Host selection tendency of key microbiota in arid desert lichen crusts**

**Running title:** Host selection tendency of lichen key microbiota

Ting-Ting Zhang^1,2^, Martin Grube^3^, Xin-Li Wei^1,2*^

^1^State Key Laboratory of Mycology, Institute of Microbiology, Chinese Academy of Sciences, Beijing 100101, China

^2^University of Chinese Academy of Sciences, Beijing 100049, China

^3^Institute of Biology, University of Graz, 8010 Graz, Austria

**Author for correspondence:**

Dr. Xin-Li Wei (Institute of Microbiology, Chinese Academy of Sciences)

Tel: 86-010-64807483

fax: 86-010-64807488

E-mail: [weixl@im.ac.cn](mailto:weixl@im.ac.cn)

Address: No. 3, No. 1 Beichen West Road, Chaoyang District, Beijing

Count of the figures: 8

**METHODS**

**Sites and sampling**

Considering different desert types related to MAP and altitude-related MAT, samples were collected from the following three deserts HNZ (Tibetan Autonomous Prefecture of Hainan), BY (Baiyin City), and YM (Yumen City). According to available specimen preservation records in HMAS-L, these three sites have a high number of *Endocarpon*. HNZ is located in the northeast of the Tibetan Plateau, with an average altitude of > 3000 m. YM is located in the northwest of Gansu Province, with mountains, plains, and Gobi terrain. BY is located in the transition zone from the Loess Plateau to the Tengger Desert. Samples were collected using sterile tweezers, stored separately in sterile polyethylene bags, and cooled on dry ice until further processing in the laboratory. The mean annual temperature and mean annual precipitation for all sampling sites were obtained from the WorldClim database ([www.worldclim.org](http://www.worldclim.org)) [1]. We subdivided *Endocarpon* crusts into four strata (Figure S6), including 1) lichen aboveground portion (Lobes, L), referring to the thallus after excluding the rhizines, 2) lichen underground portion (Rhizines, R), referring to the rhizines cut from the lichen thallus, 3) crust soil (CS), referring to the soil attached to the R within 5 mm below L, which was carefully separated from the rhizines in the lab, and 4) crust-free soil (S) located at 5 cm below L on a vertical scale which was collected directly from the field. Detailed information of the specimens and sampling sites can be found in the Supporting Information (Figure S7, S8; Table S4).

**DNA extraction, PCR amplification, and sequencing**

Soil samples (CS and S) are grinded with a mortar and pestle and pass through a 0.2 mm sieve to remove plant debris and gravels. About 0.5 g of soil sample were added directly to the PowerSoil DNA isolation kit (MoBio Laboratories Inc., Carlsbad, CA, USA) according to the manufacturer’s instructions to extract genomic DNA. The lichen samples (L and R) were surface sterilized by rinsed in 75% ethanol for 1 min, rinsed three times with sterile water. Genomic DNA was extracted using the modified CTAB method [2].

The 16S rDNA hypervariable region 4 was amplified in a 25 µl reaction mixture containing 2.5 µl of 10 × Ex Taq Buffer (20 mM Mg^2+^ plus), 0.5 µM of each primer, 2 µl dNTP (2.5 mM each), 0.5 U Ex Taq (TaKaRa, Japan), and 10 ng of template DNA. The primers used were 515F and 806R linked with 12-base barcode sequences [3]. The PCR amplification consisted of an initial denaturation step at 95°C for 5 min, 30 cycles of denaturation at 95°C for 30 s, annealing at 56°C for 40 s, and extension at 72°C for 40 s, with a final extension at 72°C for 5 min. Three replicates of each sample’s PCR products were checked by electrophoresis on 1% agarose gels and then mixed and purified using a PCR Product Purification Kit (Omega Bio-Tek, USA). NanoDrop One Spectrophotometer (Thermo Scientific, Wilmington, USA) was used to detect the quality of the purification product, and Qubit2.0 (Thermo Scientific, Wilmington, USA) was used for the accurate measurement of nucleic acid concentrations. Next, we pooled the purified PCR products with the same amount (100 ng) from each sample. Sequencing library was constructed, and pyrosequencing was performed on an Illumina NovaSeq PE250 platform at the Shanghai Personalbio Biotechnology facility (Shanghai, China). The raw sequence data in this paper are available in the NCBI Sequence Read Archive under BioProject PRJNA873937.

**Bioinformatics analysis**

All paired-end sequences were merged by *fastq_mergepairs* and quality-filtered by the *fastq_filter* command in USEARCH version 10 [4, 5]. The samples were then renamed according to metadata, and the primers were removed. We checked and deleted the potential chimeras against the SILVA (version 138) database using the *uchime* command in USEARCH. Of the remaining sequences, the redundant sequences were removed, and low-abundance reads (<8 total counts) were discarded using the *derep* command. Next, these high-quality, nonchimeric sequences at 100% using unoise2 with default parameters clustered to the zero-radius operational taxonomic units (ZOTUs) [6]. The representative sequences were assigned to taxonomic lineages using the SILVA (version 138) database at a confidence threshold of 0.8 [5, 7]. ZOTUs assigned to the chloroplast, mitochondrion, or Archaea were removed.

**Statistical analysis**

All statistical analyses were performed in R (version 4.0.5. HTTP:// www.r-project. org). Alpha diversity indices (shannon index and richness index) were calculated using USEARCH [5]. The differences among samples collected from sites and strata were evaluated using a nonparametric statistical test (Kruskal–Wallis test, Dunnett’s test). Constrained principal coordinates analysis (CPCoA) generated from Bray–Curtis distance matrixes was used to compare community similarity [5]. Permutational multivariate analysis of variance (PerMANOVA) tests were performed to determine the effects of grouping methods on community dissimilarity using the *adonis* function with 999 permutations in the vegan package [8]. Microbial community similarities were calculated by the Bray–Curtis index, while environmental distance was calculated by the Euclidean distance [9]. We selected the nine most abundant phyla and defined the remaining low-abundance phyla as “others” to demonstrate species composition with a stacked histogram. The Kruskal–Wallis test was conducted to analyze differences in community composition between collection sites, strata, and species. Simultaneously, the differences in the community composition of strata were compared according to the nutrient type of bacteria [10-12]. We performed canonical correlation analysis (CCA) to determine the relationship among environmental factors (MAP, MAT and altitude) and bacterial community structure using Monte Carlo permutation (999 repetitions)[13]. Variation partitioning analysis (VPA) was conducted to determine the relative contribution of variables to bacterial community differences using the *varpart* function with 999 permutations in the vegan package. Spatial distance between collection sites were calculated as the Euclidean distance of geographic coordinates using *pcnm* function. And the significance of the climate factors (MAP and MAT), sample attribute (strata) and spatial distance were assessed by PerMANOVA test.

We used *SourceTracker* (version 1.0) [14] based on the Bayesian community-wide culture-independent method to determine the sources of bacterial communities in each stratum. Differential abundance taxa analysis was performed using the *EdgeR* generalized linear model approach (enriched: FDR < 0.05 and log2FC >= 1, depleted: FDR < 0.05 and log2FC <= 1, no diff: FDR >= 0.05 or abs (log2FC) > 1)[15], and dissimilarity index (DSI) was defined to estimate bacterial selection processes from putative species pool to other strata [16]. Stochasticity was evaluated in the bacterial community assembly in each stratum by calculating the Raup–Crick index (RCI) and the beta nearest taxon index (βNTI) in R [17, 18].

Separate co-occurrence networks were constructed for strata to investigate differences in microbial interactions. ZOTUs with a relative abundance of >0.05% were selected, and robust correlations determined using Spearman’s correlation coefficients (*r* values) of >0.6 and false discovery rate-corrected *P* values of <0.05 were used to construct networks using the *igraph* package [19]. The following metrics of the network topological features were calculated: average degree, average path length, diameter, closeness centrality, betweenness centrality, eigenvector centrality, modularity, transitivity, robustness, and vulnerability [20, 21]. We computed nodes within-module connectivity (*Zi*) and among-module connectivity (*Pi*) to identify putative keystone species [22, 23]. Networks were visualized using the interactive Gephi platform [24].

**REFERENCES**

1. Fick, Stephen E., Robert J. Hijmans. 2017. “WorldClim 2: new 1‐km spatial resolution climate surfaces for global land areas.” *International Journal of Climatology* 37: 4302-4315. <https://doi.org/10.1002/joc.5086>

2. Rogers, Scott O., Arnold J. Bendich. 1989. Extraction of DNA from plant tissues. *Plant Molecular Biology Manual* Springer Netherlands, 73-83. <https://doi.org/10.1007/978-94-009-0951-9_6>

3. Caporaso, James A., Christian Lauber, William Walters, Donna Berg-Lyons, Catherine Lozupone, Peter Turnbaugh, Noah Fierer, Rob Knight. 2011. “Global patterns of 16S rRNA diversity at a depth of millions of sequences per sample.” *Proceedings ofthe National Academy of Sciences, USA* 108 Suppl 1: 4516-4522. <https://doi.org/10.1073/pnas.1000080107>

4. Edgar, Robert. 2013. “UPARSE: Highly accurate OTU sequences from microbial amplicon reads.” *Nature Methods* 10: <https://doi.org/10.1038/nmeth.2604>

5. Liu, Yongxin, Yuan Qin, Tong Chen, Meiping Lu, Xubo Qian, Xiaoxuan Guo, Yang Bai. 2021. “A practical guide to amplicon and metagenomic analysis of microbiome data.” *Protein Cell* 12: 315-330. <https://doi.org/10.1007/s13238-020-00724-8>

6. Edgar, Robert. 2016. 'UNOISE2: improved error-correction for Illumina 16S and ITS amplicon sequencing', bioRxiv.

7. Quast, Christian, Elmar Pruesse, Pelin Yilmaz, Jan Gerken, Timmy Schweer, Pablo Yarza, Jörg Peplies, Frank Glöckner. 2012. “The SILVA ribosomal RNA gene database project: Improved data processing and web-based tools.” *Nucleic acids research* 41: D590-596. <https://doi.org/10.1093/nar/gks1219>

8. Oksanen, Jari, Roeland Kindt, Pierre Legendre, Bob O’Hara, M. Henry H. Steven. 2007. “The Vegan Package.” *Community Ecology Package* 10: 631-637.

9. Wu, Minghu, Shengyun Chen, Jianwei Chen, Kai Xue, Shilong Chen, Xiaoming Wang, Tuo Chen, et al. 2021. “Reduced microbial stability in the active layer is associated with carbon loss under alpine permafrost degradation.” *Proceedings of the National Academy of Sciences, USA* 118: e2025321118. <https://doi.org/10.1073/pnas.2025321118>

10. Ling, Ning, Tingting Wang, Yakov Kuzyakov. 2022. “Rhizosphere bacteriome structure and functions.” *Nature Communications* 13: 836. <https://doi.org/10.1038/s41467-022-28448-9>

11. Yang, Yang, Ting Li, Yunqiang Wang, Yanxing Dou, Huan Cheng, Liangxu Liu, Shaoshan An. 2021. “Linkage between soil ectoenzyme stoichiometry ratios and microbial diversity following the conversion of cropland into grassland.” *Agriculture, Ecosystems and Environment* 314: 107418. <https://doi.org/10.1016/j.agee.2021.107418>

12. Li, Hua, Shan Yang, Mikhail V. Semenov, Fei Yao, Ji Ye, Rencang Bu, Ruiao Ma, et al. 2021. “Temperature sensitivity of SOM decomposition is linked with a K-selected microbial community.” *Global Change Biology* 27: 2763-2779. <https://doi.org/10.1111/gcb.15593>

13. Tan, Xiangping, Yanxie Nie, Xiaomin Ma, Zhiming Guo, Yang Liu, Haixia Tian, Mallavarapu Megharaj, Weijun Shen, Wenxiang He. 2021. “Soil chemical properties rather than the abundance of active and potentially active microorganisms control soil enzyme kinetics.” *Science of Total Environment* 770: 144500. <https://doi.org/10.1016/j.scitotenv.2020.144500>

14. Knights, Dan, Justin Kuczynski, Emily Charlson, Jesse Zaneveld, Michael Mozer, Ronald Collman, Frederic Bushman, Rob Knight, Scott Kelley. 2011. “Bayesian community-wide culture-independent microbial source tracking.” *Nature Methods* 8: 761-763. <https://doi.org/10.1038/nmeth.1650>

15. Robinson, Mark, Davis McCarthy, Gordon Smyth. 2010. “edgeR: A Bioconductor package for differential expression analysis of digital gene expression data.” *Bioinformatics* 26: 139-140.

16. Xiong, Chao, Yongguan Zhu, J. T. Wang, Brajesh Singh, Lili Han, Jupei Shen, Peipei Li, et al. 2021. “Host selection shapes crop microbiome assembly and network complexity.” *New Phytologist* 229: 1091-1104. <https://doi.org/10.1111/nph.16890>

17. Stegen, James, Xueju Lin, Jim Fredrickson, Xingyuan Chen, David Kennedy, Christopher Murray, Mark Rockhold, Allan Konopka. 2013. “Quantifying community assembly processes and identifying features that impose them.” *The ISME Journal* 7: 2069-2079. <https://doi.org/10.1038/ismej.2013.93>

18. Vass, Máté, Anna Székely, Eva Lindström, Silke Langenheder. 2020. “Using null models to compare bacterial and microeukaryotic metacommunity assembly under shifting environmental conditions.” *Scientific Reports* 10: 2455. <https://doi.org/10.1038/s41598-020-59182-1>

19. Csárdi, Gábor, Tamás Nepusz. 2006. “The Igraph software package for complex network research.” *International Journal of Systems Science* 1695: 1-9.

20. Zhang, Zeyu, Yang Sean Xiao, Yabin Zhan, Zengqiang Zhang, Youzhou Liu, Yuquan Wei, Ting Xu, Ji Li. 2022. “Tomato microbiome under long‐term organic and conventional farming.” *iMeta* 1: <https://doi.org/10.1002/imt2.48>

21. Yuan, Mengting Maggie, Xue Guo, Linwei Wu, Ya Zhang, Naijia Xiao, Daliang Ning, Zhou Shi, et al. 2021. “Climate warming enhances microbial network complexity and stability.” *Nature Climate Change* 11: 343-348. <https://doi.org/10.1038/s41558-021-00989-9>

22. Shi, Yu, Delgado-Baquerizo Manuel , Yuntao Li, Yunfeng Yang, Yongguan Zhu, Peñuelas Josep, Haiyan Chu. 2020. “Abundance of kinless hubs within soil microbial networks are associated with high functional potential in agricultural ecosystems.” *Environment International* 142: 105869. <https://doi.org/10.1016/j.envint.2020.105869>

23. Wang, Shang, Xiaobo Wang, Xingguo Han, Ye Deng. 2018. “Higher precipitation strengthens the microbial interactions in semi-arid grassland soils.” *Global Ecology and Biogeography* 27: 570-580. <https://doi.org/10.1111/geb.12718>

24. Bastian, Mathieu, Sebastien Heymann, Mathieu Jacomy. 2009. 'Gephi: An open source software for exploring and manipulating networks', *Third International AAAI Conference on Weblogs and Social Media*, San Jose, California, USA,.


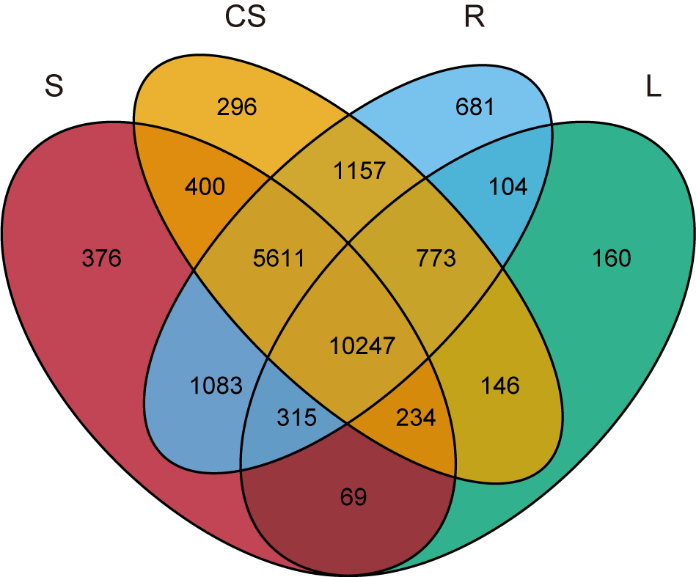


**Figure S1 Venn diagrams demonstrated the overlaps of bacterial community among the four strata (S, CS, R, L) at the ZOTU level.**


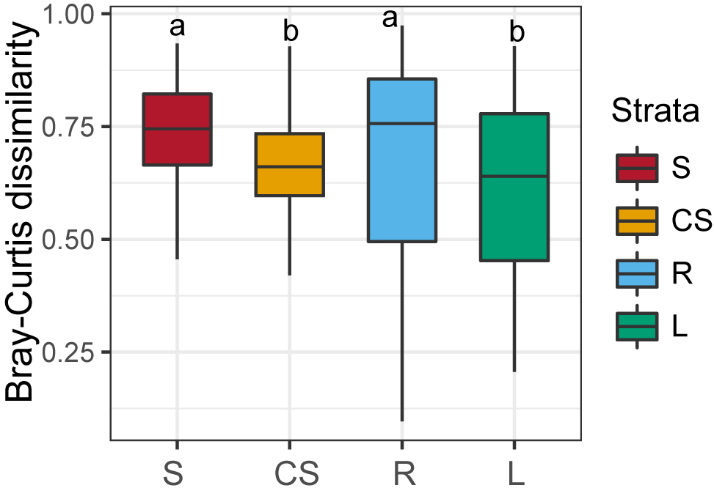


**Figure S2** **Beta-dispersion analysis based on Bray–Cutis dissimilarity.** Box plots showed the range of estimated values between 25% and 75%. The black line inside each box represents the median value. Different lowercase letters indicate significant differences between groups (alpha = 0.05, Kruskal–Wallis test).

**
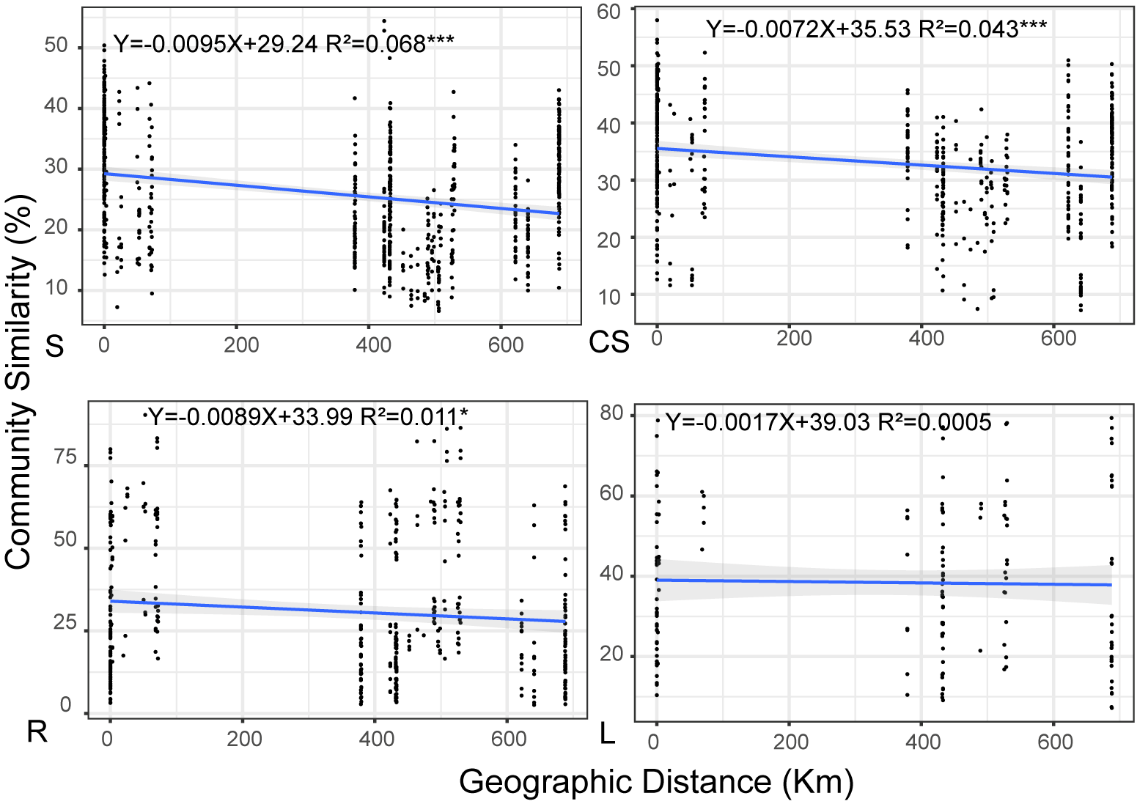
**

**Figure S3** **Relationship between geographic distance and bacterial community similarity.** Distance-decay curves showing Bray–Curtis similarity against geographic distances between four strata (S, CS, R, L). Different strata lead to the varying degrees of turnover rates of bacterial community. Asterisks indicate the statistical significance (****p* < 0.001; **p* < 0.05).

**
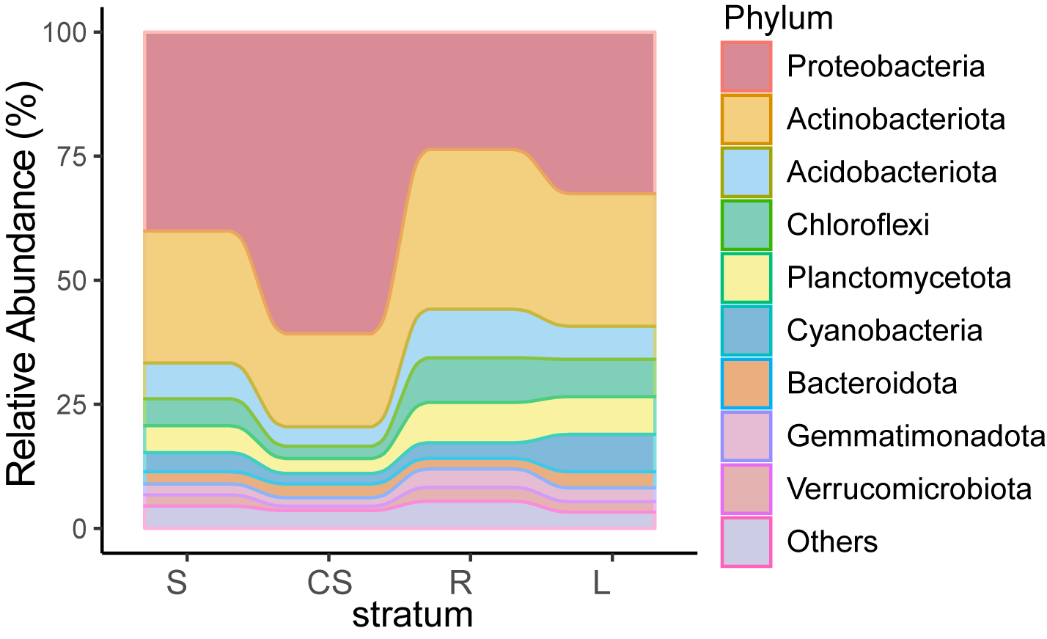
**

**Figure S4** **Dynamics of relative abundance for the top nine bacterial phyla in four strata.** Low abundance phyla are grouped into “Others”.


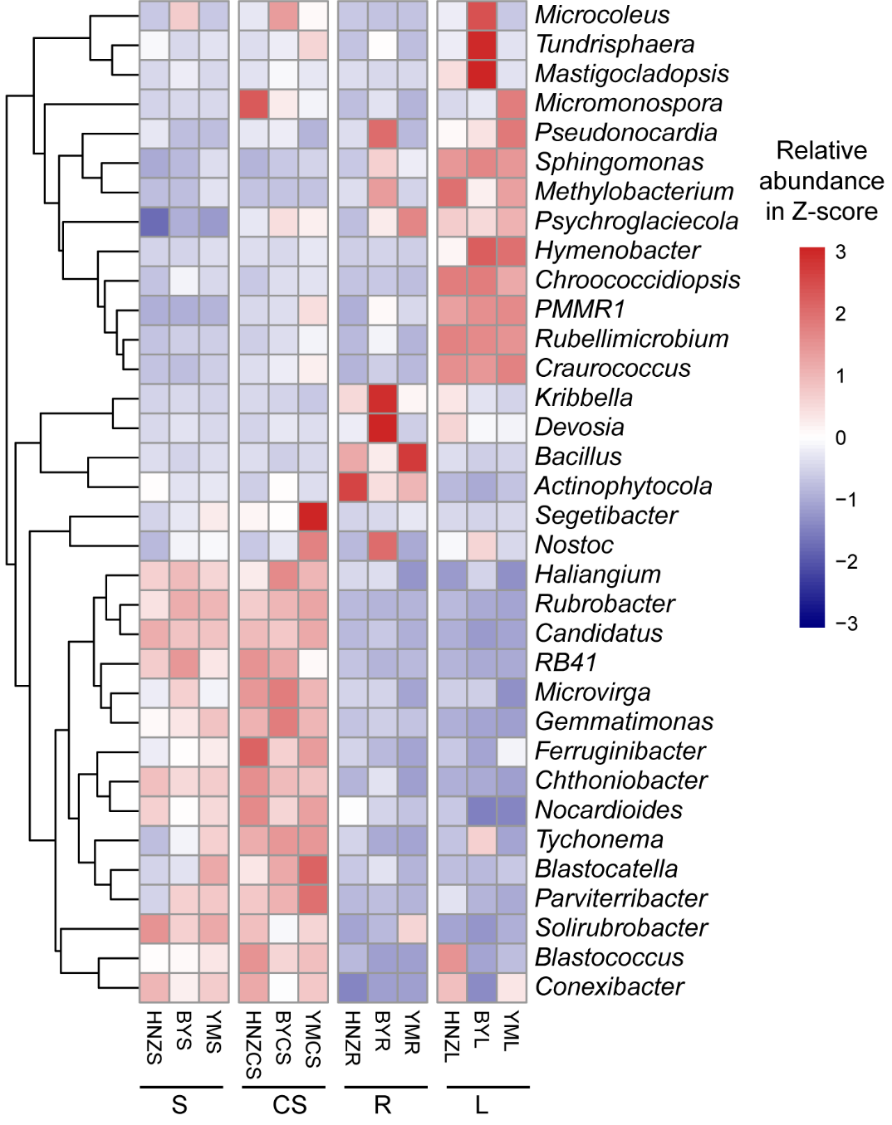


**Figure S5** **Heatmap depicting the distribution of bacterial ZOTUs (top 35 genera) in four strata.** The color of each heat map cell indicates the relative abundance of the corresponding bacterial ZOTUs treated by zero-mean normalization.


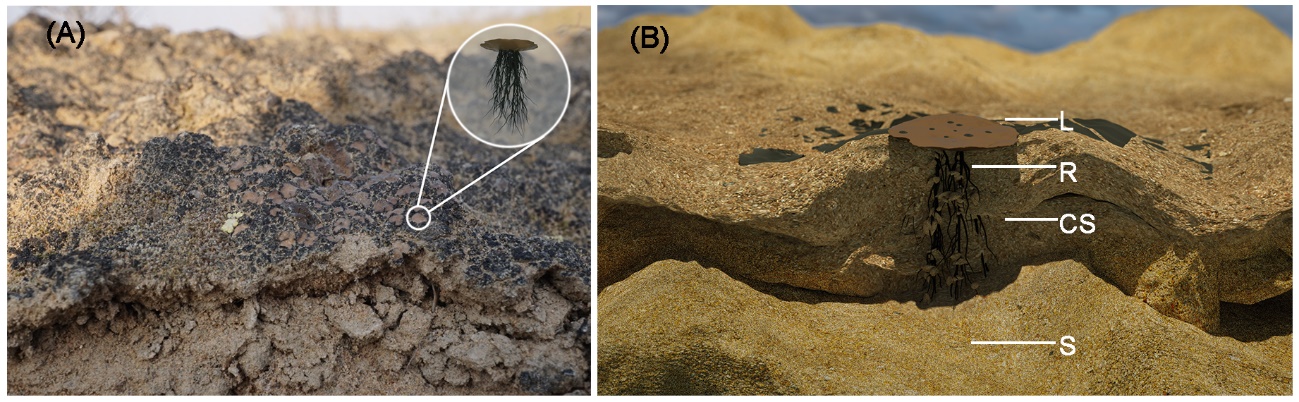


**Figure S6** **Arid desert lichen crust *Endocarpon* and the four strata.** (A) The habit of *Endocarpon* crust in the field as brown squamule on the surface of the sand soil. (B) Schematic cross-sectional view of *Endocarpon* crust and the four strata focused in this study.


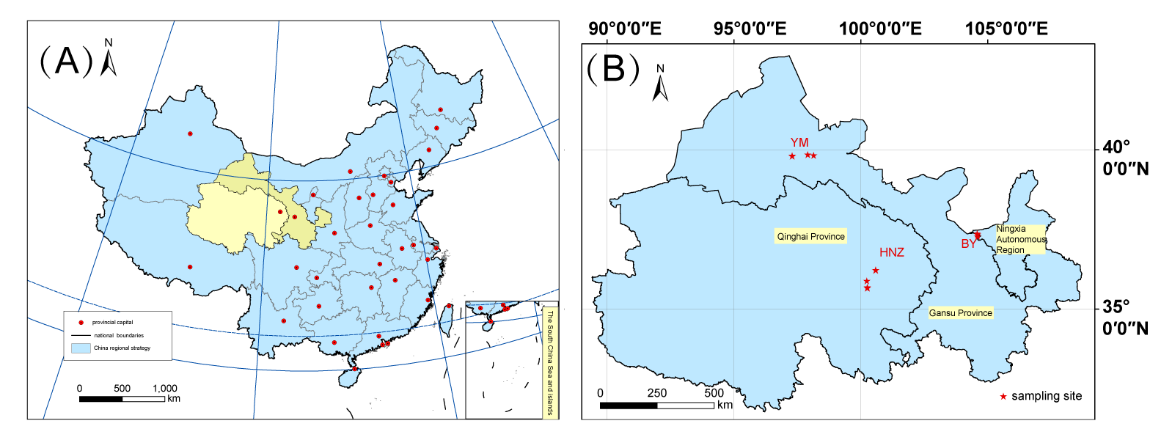


**Figure S7 The distribution of sampling sites in China.** The corresponding two provinces involved are marked with varying degrees of yellow (A), and the detailed collection areas are marked in a solid red asterisk (B). The map is based on the standard map downloaded from the standard map service website of the National Geomatics Center of China, and the base boundaries have not been modified.


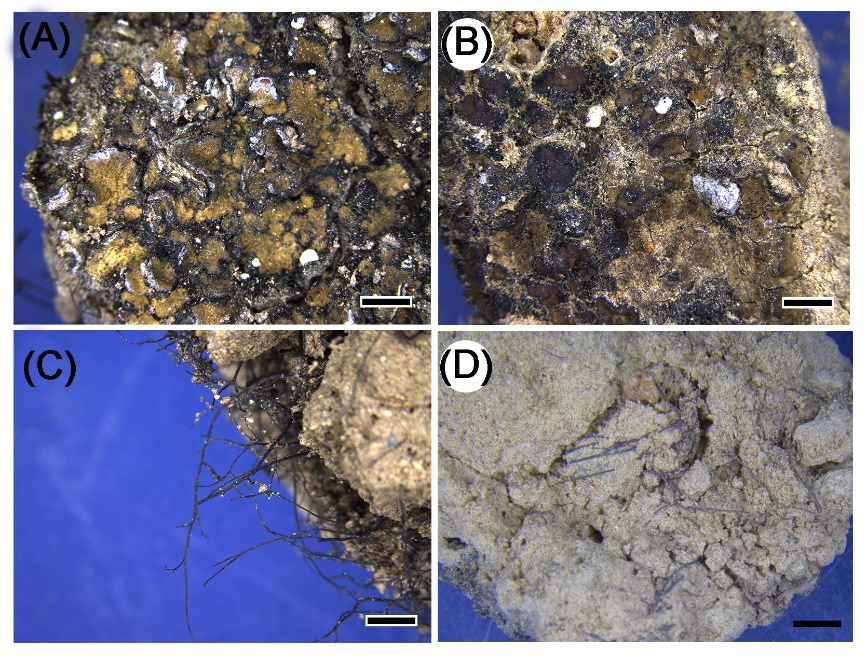


**Figure S8** **The habit of *Endocarpon* aboveground portion of squamule-form lobes (L) and underground portion of black rhizines (R).** Lichen aboveground portion of squamule-form lobes (L) of *Endocarpon* *adsurgens* (HMAS-L 154768) (A) and *Endocarpon pusillum* (HMAS-L 154767) (B). Lichen underground portion of black rhizines (R) of *Endocarpon adsurgens* (HMAS-L 154768) (C) and *Endocarpon pusillum* (HMAS-L 154767) (D). Bars: a-d = 2 mm.
